# Translational Control by Ribosome Pausing in Bacteria: How a Non-uniform Pace of Translation Affects Protein Production and Folding

**DOI:** 10.3389/fmicb.2020.619430

**Published:** 2021-01-11

**Authors:** Ekaterina Samatova, Jan Daberger, Marija Liutkute, Marina V. Rodnina

**Affiliations:** Department of Physical Biochemistry, Max Planck Institute for Biophysical Chemistry, Göttingen, Germany

**Keywords:** translation, ribosome pausing, tRNA, prokaryotes, cotranslational folding, translation efficiency, nascent peptide

## Abstract

Protein homeostasis of bacterial cells is maintained by coordinated processes of protein production, folding, and degradation. Translational efficiency of a given mRNA depends on how often the ribosomes initiate synthesis of a new polypeptide and how quickly they read the coding sequence to produce a full-length protein. The pace of ribosomes along the mRNA is not uniform: periods of rapid synthesis are separated by pauses. Here, we summarize recent evidence on how ribosome pausing affects translational efficiency and protein folding. We discuss the factors that slow down translation elongation and affect the quality of the newly synthesized protein. Ribosome pausing emerges as important factor contributing to the regulatory programs that ensure the quality of the proteome and integrate the cellular and environmental cues into regulatory circuits of the cell.

## Translation Regulation in Bacteria

Translation is an essential step in the expression of protein-coding genes, which defines the composition of the cellular proteome. Timely production of functional proteins is of central importance in maintaining cell viability, as the proteins – which comprise 60–80% of cellular biomass in bacteria – play key roles in every process in living cells. Translation is the most conserved and energy-demanding process in the cell, which consumes two-thirds of the total cellular energy during rapid growth (Russell and Cook, 1995; Klumpp et al., 2013; Basan et al., 2015). More than 40% of the protein synthesis capacity is dedicated to maintaining the translational machinery itself and in particular the ribosomes (Li et al., 2014). In bacteria, translational control ensures rapid response to changes in environmental cues, which is then followed by global changes in cell physiology, including adjustments in transcriptional profiles, alterations in ribosome biogenesis, and switching to ribosome hibernation programs. However, even under constant environmental conditions, some mRNAs are translated more often than others, resulting in a characteristic copy number of proteins synthesized from their respective mRNA, which is defined as translational efficiency (TE).

**Figure 1:**
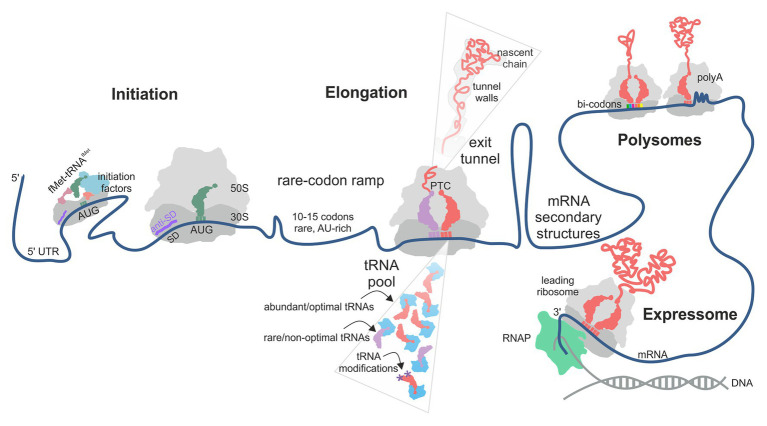
Factors contributing to translational efficiency (TE) and protein folding in bacterial translation. The schematic follows the mRNA direction from the 5' (left) to the 3' (right) end. The TE of an mRNA is largely determined at the translation initiation step when the 30S ribosomal subunit is recruited to the start codon on the mRNA. In some cases, next 30S subunit can be recruited to a stand-by site upstream of the initiation site. A rare-codon ramp of 10–15 A/U-rich codons at the beginning of the coding region can increase TE by disfavoring mRNA secondary structures at the start codon. During translation, rare codons, low-abundance aa-tRNAs, lack of tRNA modifications and the interactions of the nascent chain with the polypeptide exit tunnel of the ribosome may cause ribosome pausing. mRNA secondary structures can regulate ribosome occupancy at the upstream sequences. Some mRNA contexts, such as particular bi-codons, poly(Pro) and poly(Lys) sequences, cause rearrangements in the peptidyl transferase center (PTC) and promote formation of unusual structures in the A-site, thereby promoting ribosome stalling. Interactions between ribosomes in a polysome and of a leading ribosome with the RNA polymerase (RNAP) may provide yet another source of pausing.

In principle, translation can be regulated at any of its steps, i.e., initiation, elongation, termination, or ribosome recycling. The initiation step, at which the ribosome selects the mRNA and finds the open reading frame (ORF), to a large extent, controls the frequency at which a given mRNA is translated (Milon and Rodnina, 2012; Gualerzi and Pon, 2015; Tollerson and Ibba, 2020; Figure 1). The basal translation level is determined by the accessibility of the ribosome binding site on the mRNA, the nature of the start codon, the position of the Shine-Dalgarno (SD) sequence relative to the start codon and its complementarity to the anti-SD sequence in the 16S rRNA, and the presence of A/U rich sequences that may be specifically recognized by ribosomal protein bS1 (Gualerzi and Pon, 2015; Cifuentes-Goches et al., 2019; Saito et al., 2020). The accessibility of the ribosome binding site can change depending on the environment conditions due to ligand- or temperature-induced re-folding of the mRNA or its interactions with proteins, which regulates the protein synthesis levels of selected mRNAs and is generally referred to as riboswitches (Geissmann et al., 2009; Kortmann and Narberhaus, 2012; Breaker, 2018).

Growing evidence suggests that also the elongation step can affect TE and the quality of the corresponding protein (Rodnina, 2018; Tollerson and Ibba, 2020). At each step of translation elongation, a new amino acid is added to the growing nascent chain, and the ribosome moves along the mRNA by one codon. The rate of elongation is not uniform along the mRNA, with periods of rapid movement separated by pauses. Translation termination can contribute to the diversity of the cellular proteome by producing C-terminally extended protein isoforms due to translational readthrough of a stop codon. Finally, ribosome recycling releases the ribosomal subunits from the mRNA that has been translated, thereby maintaining the pool of ribosomes available for initiation on new mRNAs. While mechanisms of translation regulation at the initiation step are well understood, the contribution of the kinetic tuning of elongation to TE and protein quality is a complex question. Although the idea that the speed and the quality of translation are related is not new, it remained controversial due to the complexity and pleiotropic origins of the effects. In this review, we will summarize new results that shed light on ribosome pausing during elongation in bacteria and discuss the role of pauses in the control of translation efficiency and protein folding.

## Non-Uniform Rate of Translation and Translational Efficiency

Variation of translation rates may be caused by several factors (Figure 1). The mRNA may contain sequences that – under given environmental conditions – can cause pausing. These include codon choice and distribution along the mRNA (Mohammad et al., 2019), codon context (Gamble et al., 2016), specific sequences, such as poly(A) tracts (Koutmou et al., 2015), and the mRNA structure (Wen et al., 2008). The composition of the aa-tRNA pool is important as well (Rudorf et al., 2014; Vieira et al., 2016; Dykeman, 2020). The translatome of the cell, i.e., the pool of mRNA that are translated at a given moment, the tRNA charging levels, and tRNA modification efficiency may change under conditions of nutritional stress, thereby affecting the efficiency of decoding of some codons (Elf et al., 2003; Ranjan and Leidel, 2019; Dykeman, 2020). Finally, the sequence of the nascent peptide may regulate translation by, e.g., stalling translation at specific sites (Ito and Chiba, 2013; Wilson et al., 2016) or modulating translation *via* interactions of charged residues in the nascent peptide with the polypeptide exit tunnel wall of the ribosome. Ribosome pausing prolongs the time required to complete translation of a given mRNA, which – in the simplest model – could result in fewer copies of protein per mRNA per unit of time and thus a lower TE. Thus, a simple model would predict an effect of ribosome pausing on the TE, which we will discuss in detail below.

### Codon Usage

It is long known that the same protein sequence can be encoded in the mRNA in various ways using synonymous codons, which specify the insertion of the same amino acid but differ in their nucleotide composition. Synonymous codons can be read by a single tRNA, such as tRNA^Lys^ or tRNA^Phe^, which in *Escherichia coli* decode their codons AAA/AAG and UUU/UUC, respectively. However, for most four- and six-codon families, there are several tRNAs that deliver the same amino acid but differ in their decoding preferences; such tRNAs are called isoacceptor tRNAs. Isoacceptor tRNAs can usually recognize several synonymous codons through non-Watson-Crick “wobble” base pairing at the third codon position. How many and which codons can be decoded by one particular tRNA is controlled by post-transcriptional tRNA modification (Grosjean and Westhof, 2016). The rate of decoding a particular codon depends not only on the complementarity of the codon-anticodon complex but also on the concentration and properties of the respective cognate aa-tRNA in the total tRNA pool.

The preference for particular codons (codon usage bias) and their distribution along the mRNA are non-random and specific for each organism (Grantham et al., 1980; Ikemura, 1985; Moura et al., 2005). Analysis of synonymous codon frequencies in different genes has shown a bias toward usage of optimal codons in highly expressed mRNAs (Ikemura, 1981; Quax et al., 2013), which led to the suggestion that codon usage bias defines the TE, and that the presence of slowly translated codons results in lower TE (Ikemura, 1981; Sharp and Li, 1987; Dong et al., 1996; Tuller et al., 2010; Quax et al., 2013). However, later bioinformatics analyses did not support this assertion (Kudla et al., 2009; Allert et al., 2010; Subramaniam et al., 2014; Cambray et al., 2018). Expression studies found little or no correlation between codon bias and gene expression in *E. coli* using a synthetic library of 154 variants of green fluorescent protein (GFP) with random synonymous substitutions (Kudla et al., 2009), as well as a synthetic library of 285 genes (Allert et al., 2010) and 244,000 genes fused with GFP (Cambray et al., 2018) under normal conditions. In contrast, the enrichment in rapidly decoded codons in highly expressed genes becomes important under conditions of amino acid starvation, where the difference in the rate of aminoacylation of tRNA isoacceptors has a significant impact on the TE (Elf et al., 2003; Subramaniam et al., 2013, 2014; Wohlgemuth et al., 2013).

Interestingly, translation of the first 10–15 codons of ORFs appears to be special, as these stretches are enriched with non-optimal codons which are mostly A/U-rich, at least in *E. coli* (Goodman et al., 2013; Cambray et al., 2018; Verma et al., 2019; Osterman et al., 2020; Figure 1). Translation of these stretches is relatively slow (Oh et al., 2011), which would predict a lower TE, but in fact the high A/U content correlates with high TE (Moreira et al., 2019; Verma et al., 2019; Osterman et al., 2020). One possible explanation is that the A/U-rich sequences at the beginning of the ORF act as a translation enhancer (Qing et al., 2003) by reducing the stability of mRNA secondary structures around the ribosome binding site, thereby facilitating initiation and increasing TE (Gu et al., 2010; Goodman et al., 2013; Bhattacharyya et al., 2018). Alternatively, A/U-rich sequences may be preferentially recruited by the ribosomal protein bS1, which is the largest ribosomal protein and an RNA chaperone (Duval et al., 2013; Byrgazov et al., 2015). bS1 is composed of six contiguous domains known to bind to such sequences in the 5' UTR (Boni et al., 1991; Duval et al., 2013). Whether it also recruits mRNAs through the A/U sequences at the beginning of the coding region has not been tested so far.

One important question is whether rare codons are decoded more slowly than the abundant codons, thereby locally slowing down the progression of ribosomes along the mRNA, which is called ribosome pausing. This question should be readily answered by ribosome profiling, a method that enables the detailed measurements of translation *in vivo* at single codon resolution (Ingolia et al., 2009). While in mammalian and yeast cells ribosome profiling demonstrated a robust anti-correlation between codon optimality and ribosome occupancy (Weinberg et al., 2016; Wu et al., 2019), for bacteria, and *E. coli* in particular, the method has been more problematic due to technical caveats of stopping translation and alignment of ribosome-protected mRNA fragments (Oh et al., 2011; Subramaniam et al., 2014). Recent improvement of sample preparation for the ribosome profiling in *E. coli*, including rapid cell harvesting and methods to arrest translation, confirmed that also in bacteria, the ribosome density correlates with the codon-adaptation index, consistent with the expectation that rare codons are decoded by lower-abundance tRNAs more slowly than more abundant codons by their respective tRNAs (Mohammad et al., 2019), although the correlation is not as strong as in yeast cells (Weinberg et al., 2016). As a complementary method, ribosome pausing sites can be identified by the so-called integrated Nascent chain Profiling, iNP (Ito et al., 2011; Chadani et al., 2016). The approach is to isolate peptidyl-tRNAs accumulating when the ribosome pauses at a certain codon for a long time, which is defined as ribosome stalling, and identifying the sequence of the nascent peptide and its respective tRNA. Such analysis could potentially provide very accurate information on the duration of ribosome pausing but lacks so far the high-throughput character of the ribosome profiling technique.

**Figure 2:**
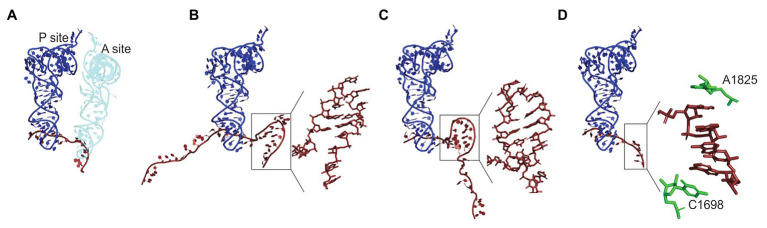
Regulation of the A-site accessibility by unconventional mRNA secondary structures formed upon ribosome pausing. **(A)** Positions of the P- (dark blue) and A-site (light blue) tRNAs on the mRNA (dark red) in *Escherichia coli* ribosome (PDB: 7K00; Watson et al., 2020). The density of the ribosome is omitted for clarity. The A-site is occupied by tRNA. In panels **(B–D)**, the A-site tRNA is absent and the P-site tRNA is used for alignment. **(B)** An mRNA element in the A-site of yeast ribosome stalled on CGA-CCG inhibitory bicodon (PDD: 6T4Q; Tesina et al., 2020). Inset: a close up of the secondary structure element. **(C)** A hairpin formed in the A-site of *E. coli* ribosome stalled on a take-off site of gene 60 mRNA of bacteriophage T4 prior to bypassing (PDB: 5NP6; Agirrezabala et al., 2017). Inset: a close up of the secondary structure element. **(D)** Mammalian ribosome stalled on a poly(A) sequence (PDB: 6SGC; Chandrasekaran et al., 2019). Inset: a close-up of the single stranded helix in the A-site. Residues A1825 and C1698 of 18S rRNA (green) stabilize helix formation by stacking.

Some aspects of the codon usage bias go beyond the simple distribution of rare and abundant codons. Bioinformatics analysis of *E. coli* genome suggests that certain codon pairs are overrepresented (i.e., codon pairs observed more frequently than predicted) in the genes coding for non-abundant proteins and underrepresented in highly expressed genes (Boycheva et al., 2003; Moura et al., 2005; Tats et al., 2008; Guo et al., 2012). The order of codons in the codon pairs is crucial; for example, the codon pair AAG-UUA is overrepresented in highly expressed genes and UUA-AAG in poorly expressed genes (Boycheva et al., 2003). Although no experimental data are available on how codon combinations may regulate the elongation rate in bacteria, translation inhibition by specific codon pairs has been demonstrated in yeast (Gamble et al., 2016). In yeast, translation of such inhibitory pairs is slower than expected for the sum of translation times for each codon individually; inhibition is abolished by replacing the sequence with synonymous codons or by reversing the codon order (Gamble et al., 2016; Tesina et al., 2020). The structure of the ribosome stalled on CGA-CCG and CGA-CGA inhibitory codon pairs showed that in both cases, the mRNA conformation in the A-site disturbs the aa-tRNA binding or accommodation, which causes the ribosome stalling (Figure 2; Tesina et al., 2020).

Another interesting phenomenon is “codon clustering,” which is found in both pro- and eukaryotes. Rare codons often occur in clusters, rather than being randomly distributed along the mRNA (Makhoul and Trifonov, 2002; Clarke and Clark, 2008; Chartier et al., 2012). In bacteria, including *E. coli*, codon pairs formed by rare codons are overused, whereas pairs formed by common codons are underused, which is not the case in eukaryotes, where this tendency is reversed (Buchan et al., 2006). Finally, some reports suggest “co-occurrence” of synonymous codons that use the same tRNA in close proximity on the mRNA coding sequence (Cannarrozzi et al., 2010; Shao et al., 2012), although the functional significance of such clustering is not understood. The complexity and strong species-specificity of overall codon distribution suggests that cells adapt their genetic programs to the needs for the protein production beyond amino acid selection.

One of the major factors that can affect protein production in the cell is the stability of the mRNA transcript. In fact, codon usage appears to be a major determinant of mRNA stability in yeast and mammals (Hanson and Coller, 2018). In *E. coli*, codon content can modulate mRNA stability (Boel et al., 2016; Cambray et al., 2018), but the mechanisms that link rare codons with the mRNA stability are likely to be different in bacteria and eukaryotes, because bacteria lack the specific Ccr4-Not complex that in eukaryotes senses the unoccupied A-site and initiates mRNA degradation by deadenylation and decapping (Buschauer et al., 2020). The precise effect of rare codons on mRNA stability in bacteria likely depends on a delicate balance between their propensity to form secondary structures and to stall ribosomes along the ORF. Both can shield the mRNA from ribonucleases, thereby extending the mRNA lifetime and increasing TE but can also reduce the initiation frequency and increase the translation time, respectively, which decreases TE (Boel et al., 2016; Cambray et al., 2018). Furthermore, slow translation elongation due to poor codon usage may deplete the pool of free ribosomes, which in turn can reduce the initiation efficiency and thereby tends to decrease TE (Cambray et al., 2018). Thus, the relationship between mRNA stability, TE, and availability of translational components may be very complex and affected not only by the codon usage in a given mRNA but also in the pool of cellular mRNAs as a whole.

Mechanistically, codon-dependent variations in the elongation rates can arise at each of the three elongation phases: during (i) aa-tRNA selection according to the mRNA codon in the A-site (decoding); (ii) peptide bond formation between the incoming amino acid and the nascent polypeptide in the peptidyl transferase center; and (iii) tRNA-mRNA translocation, which exposes the next codon in the A-site. These reactions, alone or in combination, establish the rate of elongation at a particular codon. In the following, we will summarize potential mechanisms of pausing at each of these phases.

#### Aa-tRNA Abundance

Aa-tRNA is delivered to the A-site of the ribosome in a ternary complex with EF-Tu and GTP. The rate of decoding is determined by the rate of selection of an aa-tRNA cognate to a given codon from the total pool of aa-tRNAs, followed by GTP hydrolysis by EF-Tu and accommodation of aa-tRNA in the peptidyl transferase center of the ribosome (Rodnina, 2018). For every single codon, there is a different distribution of cognate, near-cognate, and non-cognate aa-tRNAs, with their different decoding properties, such as the number and geometry of mismatches in the codon-anticodon complex, and aa-tRNA concentrations. The competition between the aa-tRNAs results in variations in decoding times for different codon-aa-tRNA pairs (Rudorf et al., 2014; Vieira et al., 2016; Dykeman, 2020), even though the rates of reactions on the decoding pathway, such as binding, GTP hydrolysis, and tRNA accommodation, are similar for different cognate aa-tRNAs (Rodnina et al., 2005; Ledoux and Uhlenbeck, 2008). Although in bacteria, global codon usage matches the tRNA abundance, transient changes in the transcriptome composition due to transcriptional responses may shift mRNA codon bias relative to tRNA concentrations, which is predicted to have strong effects on decoding and may lead to additional, unexpected ribosome pauses (Rudorf, 2019; Dykeman, 2020). Under conditions of rapid growth, tRNAs are almost fully charged (Dittmar et al., 2005). Depletion of amino acids, e.g., during starvation, results in selective aa-tRNA charging levels for some tRNA isoacceptors, with some becoming low and others remaining high (Elf et al., 2003). Codons read by isoacceptors that retain high charging can be used for efficient translation of genes that are essential during amino-acid starvation (Dittmar et al., 2005; Figure 1).

In this context, modifications at the tRNA anticodon region play an important role, and their absence can lead to ribosome stalling at the respective codons (Ranjan and Leidel, 2019). In particular, the positions 34 and 37 in the tRNA anticodon stem-loop (ASL) affect several elongation steps, including aa-tRNA selection and translocation. Modifications at both positions affect the structure and conformational dynamics of the ASL and provide chemical groups for the non-canonical interactions with the mRNA (Agris, 2008). The modifications at position 37 are mainly associated with reading frame maintenance (Agris et al., 2018; Hou et al., 2019). The N6-threonylcarbamoyladenosine-(t6A) modification, which is commonly found at position 37 of Arg-, Asn-, Ile-, Lys-, Met-, and Ser-tRNAs, is essential for the viability of *E. coli* (Juhling et al., 2009; Thiaville et al., 2015); t6A-deficient yeast strains are viable but have increased protein aggregation due to global mistranslation (Pollo-Oliveira et al., 2020). The modifications at position 34 are particularly important as modulators of wobble-position decoding. For example, the 5-oxyacetic acid (cmo5) modification at U34 expands the decoding capacity of tRNA allowing to decode all four synonymous codons for Ala, Ser, and Val (Agris et al., 2018) and expands the codon recognition by wobble interaction toward unusual base pairing (Lim and Curran, 2001; Agris et al., 2007; Grosjean and Westhof, 2016). Ser-tRNA(UGA), carrying the cmo5 modification at U34, can form stable complexes with all four codons, adopting specific conformations to stabilize each type of interaction (Weixlbaumer et al., 2007). Another cmo5-modified tRNA, Ala-tRNA(UGC), can read the non-Watson Crick synonymous codon GCC correctly, using a U:C base pairing at the third codon position, albeit slower than codons with a conventional wobble base pairing (Kothe and Rodnina, 2007). The lack of the 5-methoxycarbonylmethyl-2-thiouridine (mcm5s2U34) modification in tRNA^Lys^, tRNA^Glu^, and tRNA^Gln^ in yeast results in ribosome queuing at the respective codons, affects expression of a subset of genes enriched for AAA, CAA, and GAA codons and leads to protein aggregation (Rezgui et al., 2013; Zinshteyn and Gilbert, 2013; Nedialkova and Leidel, 2015; Ranjan and Rodnina, 2016). Although some of these specific examples are derived from yeast, it is likely that in *E. coli*, the effect of these modifications is similar, because the basic mechanisms of codon-anticodon recognition are universally conserved in pro- and eukaryotes. In summary, the decoding properties, concentrations, and chemical modifications of aa-tRNA play an important role during decoding and may contribute to translational pausing and the overall TE of the gene.

#### Amino Acid Composition of the Nascent Peptide

Experiments with model substrates show that the rate of peptide bond formation depends on the nature of the amino acids acting as peptide donors and acceptors (Wohlgemuth et al., 2008; and earlier papers cited therein). However, for many aa-tRNAs, the chemistry step is rate-limited by the preceding step of aa-tRNA accommodation in the peptidyl transferase center on the large ribosomal subunit, which is similar for various aa-tRNAs (Wohlgemuth et al., 2008). One exception is the peptide bond formation involving consecutive Pro residues, which is much slower than with other amino acids. The rate of Pro codon translation is context-dependent with the time of pausing modulated by the preceding or following amino acid (Peil et al., 2013; Woolstenhulme et al., 2015; Mohammad et al., 2019). In bacteria, peptide bond formation between consecutive Pro residues is accelerated by EF-P (Doerfel et al., 2013; Ude et al., 2013), which helps to orient and stabilize the reactants in the peptidyl-transferase center (Doerfel et al., 2015; Huter et al., 2017). Nevertheless, even in the presence of EF-P, ribosomes pause on sequences presenting a combination of Pro, Gly, and Asp codons (Mohammad et al., 2019). Pausing on poly-Pro stretches may regulate the amount of the respective proteins in the cell, as suggested for the Cad module, a lysine-dependent acid-resistance stress-response system. The membrane-integrated sensor CadC regulates induction of the cadBA operon encoding the lysine/cadaverine antiporter CadB and the lysine decarboxylase CadA. Activation of wild-type CadC requires two stimuli, low pH and exogenous lysine, to induce cadBA expression; in the absence of exogenous lysine, CadC is inhibited by its co-sensor, lysine permease (LysP). CadC gene contains a stretch of five Pro codons, which are normally translated with the help of EF-P. In the absence of functional EF-P, the ribosome is stalled at the poly-Pro track, which reduces the copy number of CadC and abolishes the expression of the downstream cadAB operon, resulting in an inactivation of the module. In contrast, introducing mutations into the poly-Pro track results in a higher TE of all proteins encoded by the operon and makes the system less dependent on both external stimuli due to a change in the ratio between CadC and LysP. This example illustrates the potential regulatory role of EF-P and the poly-Pro stretches in fine-tuning protein expression (Ude et al., 2013).

Ribosome pauses can also be caused by the interactions of the newly synthesized nascent peptide with the walls of the polypeptide exit tunnel *via* distortion of the optimal geometry at the peptidyl transferase center (PTC; Ito and Chiba, 2013; Wilson et al., 2016). This phenomenon is best illustrated by the so-called “arrest peptides” such as SecM, MifM, VemP, and ErmCL (Ito and Chiba, 2013). These sequences are about 20 amino acids long. They cause PTC distortion through the interaction with the polypeptide exit tunnel of the ribosome, which results in the reduced rate of the peptide bond formation and ribosome stalling when slowly reacting amino acids are in the A and P sites of the PTC (such as proline and glycine; Woolstenhulme et al., 2013; Wilson et al., 2016; Seip et al., 2018; Mohammad et al., 2019).

Peptidyl transferase center also becomes distorted during translation of poly(A) sequences into stretches of poly-Lys both in *E. coli* and eukaryotes (Koutmou et al., 2015; Chandrasekaran et al., 2019). The ribosomes tend to stall and shift the reading frame when they encounter poly(A) sequences longer than 9 nucleotides (nt). The inhibition of translation, as shown for mammalian ribosomes, is due to conformational changes in both PTC and the decoding center (Chandrasekaran et al., 2019). Poly-Lys in the polypeptide exit tunnel stabilizes the PTC in a conformation that is inhibitory to peptide bond formation. In parallel, the poly(A) stretch of the mRNA adopts a single-stranded helix conformation in the decoding center, which is stabilized by the interactions with the rRNA (Figure 2). The reconfigured decoding center disfavors aminoacyl-tRNA binding to the A-site, thereby hindering the elongation even further (Chandrasekaran et al., 2019; Tesina et al., 2020). Given the high degree of evolutionary conservation of the functional centers of the ribosome and the consistent stalling of *E. coli* ribosomes by poly-Lys nascent peptide, same mechanism is likely to operate in bacteria as well.

In addition to the specialized stalling sequences, also shorter patches of amino acids may have an impact on the TE. Statistical analysis of ~6,000 genes from different organisms expressed in *E. coli* showed that the amino acid identity has a significant impact on protein expression. Bioinformatics analysis of *E. coli* proteome revealed that several amino acid triplets (for example, CMY, MWC, GPP, and WMC) and thousands of quadruplets (for example, CMYW) are either completely absent or are several-fold less abundant than expected for random sequences (Navon et al., 2016). Single-molecule FRET experiments suggested that the presence of such sequences in the mRNA increases the elongation time (Navon et al., 2016). This indicates that ribosomes tend to stall while translating these codon combinations, but the exact reason for stalling is not yet known.

The amino acid charge distribution along the nascent chain may affect translation elongation as well (Lu and Deutsch, 2008; Tuller et al., 2011). Positively charged amino acids upstream of the A-site codon slow down the ribosome irrespective of codon identity or codon distribution (Lu and Deutsch, 2008; Charneski and Hurst, 2013; Sabi and Tuller, 2015), and there is a linear correlation between the extent of ribosome pausing and the length of positively charged segments (Charneski and Hurst, 2013). The effect of charged amino acids on translation also depends on its location in the nascent peptide. Codons encoding for positively and negatively charged amino acids are non-randomly distributed along the mRNA with higher frequency of positive charges at the beginning and negative charges at the end (Duc and Song, 2018). This observation led to the suggestion that the incorporation of positively charged amino acids at the beginning of elongation helps the N-terminus of the nascent polypeptide to move toward and enter the negatively charged part of the exit tunnel (Duc and Song, 2018). Once the N-terminus passed the exit tunnel, the mean elongation rate increases with the hydrophobicity of the nascent chain (Duc and Song, 2018). While some of these studies have been conducted using yeast as a model organism (Charneski and Hurst, 2013; Sabi and Tuller, 2015; Duc and Song, 2018), the tendencies observed in yeast may be common for bacteria as well, because residues lining the exit tunnel are highly conserved in the zone proximal to the PTC and diverge only around the vestibule zone (Duc et al., 2019). Mechanistically, the effects of interactions of the nascent peptide with the exit tunnel are still poorly understood. Nascent peptide interactions with the ribosome may provide a new regulatory mechanism for sequence-specific modulation of translation speed.

#### mRNA Structure and tRNA-mRNA Translocation

The nucleotide sequence of the mRNA determines the potential secondary structure, which may create obstacles when the ribosome moves along the mRNA during the translocation phase (Figure 1). The coding regions of mRNA generally appear more structured than non-coding ones, but the structures are dynamic (Del Campo et al., 2015). Ribosome pausing at mRNA structures is likely transient due to the intrinsic helicase activity of the ribosome that can unwind thermodynamically stable mRNA structures (Takyar et al., 2005; Qu et al., 2011). The TE of ORFs within a polycistronic mRNAs negatively correlates with increasing levels of mRNA structure (Burkhardt et al., 2017). However, probing of RNA secondary structure with the so-called selective 2' hydroxyl acylation analyzed by primer extension (SHAPE) method in conjunction with parallel analysis of RNA structure (PARS) shows that only the mRNA structures at the initiation site are important and there is no significant correlation between mRNA structure in the coding region and TE (Del Campo et al., 2015; Mustoe et al., 2018). Stable *in vivo* mRNA structures mostly represent evolutionarily conserved functional elements, which serve as signals for programmed recoding events, such as frameshifting and bypassing (Rouskin et al., 2014), or for membrane insertion of inner membrane proteins (Del Campo et al., 2015). For example, mRNA structures that stimulate frameshifting can reduce the rate of translation by as much as 10-fold (Wen et al., 2008; Chen et al., 2013, 2014; Caliskan et al., 2014, 2017; Kim et al., 2014; Bock et al., 2019) by inhibiting the dissociation of deacylated tRNA from the E-site (Chen et al., 2013; Caliskan et al., 2014). On the other hand, the overall effect of mRNA structure on pausing may be counterbalanced by the selection of highly abundant codons participating in structure formation (Gorochowski et al., 2015). Vice versa, in bacteria, unstructured mRNA regions are often comprised of codons decoded by low abundance tRNAs. This, in general, can smoothen out the variation in global translation rates (Gorochowski et al., 2015). Of note, also mRNA interactions with the rRNA were suggested to slow down translation, in particular through the interactions of SD-like sequences in the mRNA coding region with the anti-SD sequence in the 16S rRNA (Li et al., 2012). Early ribosome profiling experiments seemed to support this notion (Li et al., 2012). However, more recent work using optimized ribosome profiling protocols has shown that the respective accumulation of mRNA reads was caused by technical problems and that there is no indication for pervasive ribosome pausing caused by potential SD-like sequences (Martens et al., 2015; Chadani et al., 2016; Mohammad et al., 2016, 2019). This result is consistent with the lack of peptidyl-tRNA accumulation which would be expected if the ribosome were stalled at SD-like sequences (Chadani et al., 2016).

In addition to large mRNA secondary structure elements that can modulate stalling, recent data suggest that local mRNA structures may have an unexpected effect that would be difficult to predict based on the thermodynamic stabilities of the respective structures alone (Figure 2). Pausing at these unusual structures is regulated by the interactions of the nascent peptide with the exit tunnel. As mentioned above, poly(A) stretches can form a single-stranded helix in the A-site, which is stabilized by the rRNA in the decoding region and coincides with the formation of the catalytically inactive conformation of the PTC (Figure 2D; Chandrasekaran et al., 2019; Tesina et al., 2020). Similarly, CGA-CCG and CGA-CGA sequences form secondary structures in the A-site that inhibit aa-tRNA binding (Figure 2B; Tesina et al., 2020). The gene 60 mRNA of the bacteriophage T4 codes for a sequence that can stall its own translation at a very specific codon due to interactions of the nascent peptide with the exit tunnel. The interaction induces an inactive PTC conformation and the formation of a small mRNA hairpin in the A-site (Figure 2C; Agirrezabala et al., 2017). Thus, nascent-peptide-mediated formation of local mRNA structures is an emerging new mechanism for regulation translational pauses both in bacteria and eukaryotes.

### Coupling of Transcription and Translation, Polysomes, and Ribosome Collisions

In many bacteria, including *E. coli*, transcription and translation are coupled. The mRNA emerging from the RNA polymerase (RNAP) is recruited by the leading ribosome that resides in close proximity or is even physically linked to RNAP (Landick et al., 1985; Burmann et al., 2010; Proshkin et al., 2010; Kohler et al., 2017; O’Reilly et al., 2020; Wang et al., 2020; Webster et al., 2020), forming the so-called expressome (Kohler et al., 2017; Figure 1). The rates of transcription and translation in the expressome are coordinated and change together in response to growth conditions (Vogel and Jensen, 1994). The coordination between RNAP can be either direct, i.e., *via* the bridging proteins (Burmann et al., 2010; Proshkin et al., 2010), or indirect, e.g., through second messengers such as ppGpp (Chen and Fredrick, 2020). Regardless of the coupling mechanism, the leading ribosome cannot move faster than the RNAP does, so that the pausing of RNAP may dictate the behavior of the leading ribosome (Vogel and Jensen, 1994; Proshkin et al., 2010). Surprisingly, however, it is rather the ribosome that appears to modulate the pace of RNAP. For example, acceleration or deceleration of translation by antibiotics or mutations in ribosomal proteins result in corresponding changes in the speed of RNAP (Proshkin et al., 2010). Even more remarkable, the rate of transcription correlates with the number of rare codons in a gene, presumably by slowing down the progression of the ribosome along the mRNA (Proshkin et al., 2010). How exactly the coordination between the ribosome and RNAP affects the TE for a given protein is not clear, but it clearly provides a mechanism by which bacteria can globally react to changes in growth rate and environmental conditions. One surprising aspect of the tight coupling between the RNAP and the leading ribosome is that such hand-over of the emerging mRNA transcript might prevent formation of the potential regulatory secondary structures. To be operational, such structures would have either to delay the progression of the leading ribosome relative to the RNAP or to form behind the leading ribosome. Interestingly, a recent paper suggests that coupled RNAP-ribosome movement is not a general hallmark of bacterial gene expression; rather, in some bacteria transcription is much faster than translation (Johnson et al., 2020). This finding would predict a much larger contribution of mRNA elements, such as riboswitches and secondary structure elements, in translation regulation of bacteria with “runaway” transcription, because a delay in ribosome movement would allow the emerging mRNA structures to fold. Similar regulatory mechanisms may also become more pervasive in bacteria with the coupled transcription-translation once the first round of translation is completed.

Another interesting, but yet poorly studied aspect of translation regulation is polysome formation. In exponentially growing *E. coli* cells, 70% of the ribosomes are found in polysomes (Jacobson and Baldassare, 1976). Ribosomes can load onto the mRNA in intervals of 1–3 s (Kennell and Riezman, 1977; Mitarai et al., 2008). The average polysome packing density is 1.3 ribosomes per 100 nt of mRNA, which yields an average ribosome spacing of 77 nt (Siwiak and Zielenkiewicz, 2013), with examples ranging from one ribosome every 72 nt on *luc* mRNA (Brandt et al., 2009) to one in every 100 nt on *lacZ* mRNA (Kierzek et al., 2001; Mitarai et al., 2008). Polysome formation may increase TE by protecting mRNA from degradation and thereby increasing its lifetime for producing more protein from the same mRNA or by affecting the accessibility of the ribosome binding site at the initiation step of translation (Andreeva et al., 2018). Obviously, increasing the number of ribosomes translating simultaneously the same mRNA should also increase TE. However, too high ribosome loading in a polysome, in combination with stalling of the leading ribosome, may lead to ribosome queuing which tends to reduce TE by slowing down translation after the stalling ribosome (Sorensen and Pedersen, 1991; Mitarai et al., 2008). One extreme form of such queueing are ribosome collisions, which in eukaryotes, elicit cotranslational degradation of both mRNA and nascent peptide, thereby affecting TE through a degradation pathway (Simms et al., 2017; Schuller and Green, 2018; Joazeiro, 2019; Collart and Weiss, 2020). While less is known about the ribosome collisions in bacteria, one key component of the sensing machinery that identifies stalled ribosomes, Rqc2, has a bacterial homolog, RqcH, which recognizes obstructed 50S subunits and promotes nascent chain proteolysis (Lytvynenko et al., 2019). In contrast to eukaryotic Rqc2, RqcH directly marks nascent chains for degradation by appending C-terminal poly-Ala tails that act as degrons recognized by the ClpXP protease (Lytvynenko et al., 2019). We note that it is not clear whether ribosome collisions are the only trigger of the ribosome quality control mechanism in bacteria or it is yet another general mechanism to cope with incomplete translation, overlapping with the functions of tmRNA, ArfA, and ArfB systems that sense and discard ribosomes stalled on truncated mRNAs. Thus, translation can be modulated not only by mRNA elements but also by RNAP or ribosomes bound to a given mRNA transcript, which can also attenuate the strength of the regulatory signals encoded by the mRNA.

## Non-Uniform Elongation as a Timer for Protein Folding

**Figure 3:**
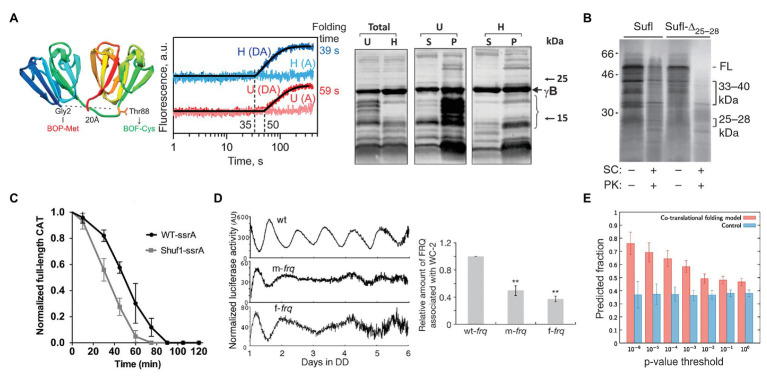
Examples of how rare codons can affect protein folding and function. **(A)** Gamma-B crystallin (Buhr et al., 2016). Left panel: Crystal structure of bovine gamma-B crystallin (PDB: 4GCR). Indicated are the protein positions used to introduce FRET labels. Middle panel: Cotranslational folding of un-optimized (U) and harmonized (H) gamma-B crystallin monitored in real time. The translation times, indicated by the delay in the time courses, are 35 s for the H and 50 s for the U construct; the delay coincides with the emergence of the N-terminal domain of gamma-B crystallin from the exit tunnel of the ribosome. The folding times, derived from exponential fitting, are 39 s for the H and 59 s for the U construct, demonstrating that folding kinetics is affected by synonymous codon replacements. Right panel: Expression of U and H proteins in *E. coli*. Shown are the total protein (Total) for the U and H variants as well as soluble (S) and pellet (P) fractions for each construct. **(B)** Stability of an *E. coli* protein Sufl (Zhang et al., 2009). Comparison of the proteinase K sensitivity of the wild-type wt Sufl and SufI-Δ25–28, in which two rare Leu codons at positons 244 and 252 were substituted by abundant codons. **(C)** Stability of the *E. coli* chloramphenicol acetyltransferase (CAT; Walsh et al., 2020). The CAT activity was measured in a ClpXP degradation assay of ssrA-tagged wt CAT and a ssrA-tagged Shuf1-CAT variant. In Shuf1-CAT, the pattern of synonymous codons is changed locally, but the global codon usage was largely unchanged. **(D)** Activity of the circadian clock protein FRQ (Zhou et al., 2013) tracked *via* Luciferase (Luc) reporter assay. Right panel: Effect of codon optimization of the circadian rhythm. m-frq, rare codons were optimized; f-frq, all codons were optimized. Left panel: Comparing periodicity and expression levels of wt and optimized FRQ variants. **(E)** Analysis of 500 *E. coli* proteins determining the fraction of conserved, slowly translated, rare codon-enriched regions that account for the predicted intermediates in a cotranslational folding model (orange) vs. changing threshold value of *p* (Jacobs and Shakhnovich, 2017). Randomized control sequences are shown in blue. **p < 0.01.

Ribosome pausing may have evolved not only to regulate the production of a given protein but also to ensure the quality of synthesized proteins (Komar, 2009; Chaney and Clark, 2015). There is growing evidence that synonymous substitutions of natural rare codons or alterations in rare tRNA abundance changes the kinetics of translation and increases protein aggregation and susceptibility to protease digestion (Komar et al., 1999; Cortazzo et al., 2002; Zhang et al., 2009; Buhr et al., 2016; Konczal et al., 2019; Walsh et al., 2020; Figure 3). A study of a gamma-B crystallin, a mammalian eye-lens protein, demonstrated that synonymous codon substitutions change the kinetics of translation and the lifetime of translation intermediates (Figure 3A; Buhr et al., 2016). NMR and proteolysis assays showed that this change in translation kinetics and cotranslational folding trajectory alters protein stability, solubility, and conformation (Figure 3A; Buhr et al., 2016). Similar changes in the cotranslational folding trajectory, protein function, and stability were observed for a number of other proteins, such as Sufl (Figure 3B; Zhang et al., 2009), chloramphenicol acetyltransferase (CAT; Komar et al., 1999; Walsh et al., 2020; Shuf1 in Figure 3C), or the *Echinococcus granulosus* fatty acid binding protein1 (EgFABP1; Cortazzo et al., 2002). It seems that cotranslational protein folding may be one of the main driving forces that preserve the non-uniform rate of translation through maintaining the codon usage bias.

A number of well-documented cases in eukaryotic organisms comprehensively demonstrate how synonymous mutations result in specific phenotypes (Kimchi-Sarfaty et al., 2007; Xu et al., 2013; Kim et al., 2015). A silent mutation of Ile codon AUC to a rare AUU in the coding sequence of the human MDR1 protein alters translation speed and affects the timing of cotranslational folding, resulting in a protein that has an altered conformation and a changed affinity to its substrates (Kimchi-Sarfaty et al., 2007). The change in protein function alters the pharmacokinetics of individuals carrying this silent mutation and is associated with adverse therapeutic outcomes in breast cancer patients (Cizmarikova et al., 2010). Another example is a protein from *Neurospora crassa* that functions as part of the circuit establishing the molecular circadian rhythm (Zhou et al., 2013). The gene coding for protein FREQUENCY (FRQ) has multiple regions with non-optimal codons, read by low abundance tRNAs. When codon usage is optimized, the total amount of translated protein is high, but the circadian rhythm is changed (Figure 3D; Cheng et al., 2001), indicating that the codon-optimized protein is unable to interact with its partners due to its altered structure (Zhou et al., 2013). In cyanobacterium *Synechococcus elongatus*, the molecular circadian clock proteins KaiBC also have clusters of non-optimal codons. When the coding sequences are optimized, the periodicity of expression remains unchanged compared to the wild-type protein under optimal growth conditions (at 30°C; Xu et al., 2013). However, when the temperature is lowered to 18–25°C, the codon-optimized KaiBC strains grow significantly slower than the wild-type cells (Xu et al., 2013). It is tempting to speculate that the difference may originate from changes in translation kinetics and cotranslational folding at lower temperatures. While at higher temperatures, KaiBC might be able to refold to its functional form post-translationally, at lower temperatures, this is no longer possible, perhaps due to an overload of the chaperone networks in the cold.

Membrane proteins were shown to have multiple pauses during translation *in vivo* (Chadani et al., 2016). They have long clusters of rare codons that may play a role in defining the time window for correct targeting and membrane insertion of these proteins (Zalucki and Jennings, 2007; Chartier et al., 2012; Mercier et al., 2020). The topology of transmembrane helices generally follows the “positive-inside” rule, according to which the distribution of positively charged residues at the N-terminus of the bacterial inner membrane proteins determines the orientation of the first transmembrane helix with its N-terminus in the cytoplasm or the periplasm (von Heijne, 1989). However, computer simulations suggested that variations in the translation rate may alter transmembrane helix topology (Zhang and Miller, 2012; Niesen et al., 2017), indicating that the “positive-inside” rule is not the only driver of correct membrane insertion. In fact, a recent biochemical study which monitored cotranslational insertion of transmembrane segments of *E. coli* EmrD protein, showed that a prolonged ribosome stalling at a critical position can alter the topology of transmembrane segments (Mercier et al., 2020), underscoring the important link between the rate of translation and protein biogenesis and folding.

The primary effect of ribosome pauses on protein folding is likely cotranslational, arising as the nascent peptide moves through the exit tunnel and emerges from the ribosome. Many types of folding events occur in the micro- to millisecond time scale, whereas the rate of translation in bacteria is about 10–20 amino acids/s. Thus, rapid folding events that are much faster than amino acid addition to the nascent chain occur at quasi-equilibrium conditions (O’Brien et al., 2014; Liutkute et al., 2020). So why should further slowing down translation at particular positions affect protein folding? It is known that the distribution of rare codons with respect to the protein structure is non-random. Amino acids that are buried within the protein structure and are key to the overall stability of a protein are generally encoded by optimal codons (Zhou et al., 2009). In large proteins, clusters of rare codons are found every 125–155 amino acids and mark domain boundaries (Makhoul and Trifonov, 2002; Zhang et al., 2009). Slowing down translation at these critical junctions would provide the domains with enough time for correct folding and prevent inter-domain aggregation (Makhoul and Trifonov, 2002; Zhang et al., 2009; Vasquez et al., 2016). Notably, it is the rare-codon cluster positions, rather than the codon identity that are conserved within homologous coding sequences across eukaryotic, bacterial, and archaeal species (Chaney et al., 2017), which provides a basis for rational design of genes for heterologous expression and increasing protein solubility (for review, see Komar, 2016). Molecular dynamics simulations indicate that conserved rare codons are associated with cotranslational folding intermediates that may be even smaller than protein domains, and that this mechanism is conserved across multiple prokaryotic species (Figure 3E; Jacobs and Shakhnovich, 2017). Computer simulations that estimate the cotranslational folding as a function of translation rate suggest that the extent of domain folding is defined by the states populated early during synthesis and that this entire process can be derailed by single synonymous codon substitutions (O’Brien et al., 2012). Taking into account that ribosome pausing can be caused not only by rare codons, but also by a variety of different factors described above, or even emerge due to stochastic variations in elongation transit times, the link between translation and ribosome pausing may be an important factor for defining cotranslational protein folding.

Finally, alterations in translation rate can also affect the concerted binding of cofactors and proteins involved in nascent-chain processing (Nissley and O’Brien, 2014). Disturbing the recruitment of chaperones (TF, DnaK, and GroEL) and other ribosome-associated factors has been shown to hinder protein biogenesis in eukaryotes (Zhang et al., 2010), and SRP-mediated targeting in prokaryotes (Zalucki and Jennings, 2007) and eukaryotes (Pechmann et al., 2014). *In vivo* the specific kinetics of translation and the associated trajectories of cotranslational folding is aided by the entire proteostasis network, including chaperones and the protein degradation machinery, to produce functional proteins (Mogk et al., 2011; Collart and Weiss, 2020). Any changes in the kinetics of translation that affect either the overall protein fold or the way how the protein quality control machinery interacts with nascent proteins may cause visible phenotypic outcomes (Walsh et al., 2020).

High translation speed might not always be preferable if it comes at the expense of the protein’s quality in terms of solubility, stability, and function. Rare codons and other sources for ribosome pausing at specific points of the coding sequence may be under selective pressure to keep translation slower and less efficient but generate proteins that are functionally active and structurally stable throughout their lifetime. Thus, ribosome pausing emerges as a mechanism that allows cells to balance translational efficiency with correct protein folding and thus achieve optimal fitness under given environmental conditions.

## Concluding Remarks

In recent years, it became increasingly clear that beyond the sequence information mRNA contains regulatory signals that attenuate translation and affect TE and protein folding. Although the importance of ribosome pausing is clearly established, many mechanistic questions remain open. For example, it is not clear whether only a fraction of ribosomes pause while others continue translation; what is the structure of pausing ribosomes; and what causes them to resume translation. Among the most interesting novel findings are sequences that stall translation through combined effects on the mRNA structure and on the conformation of the PTC. As the mRNA structures that fold in the A-site appear specific for each case studied, it would be interesting to see more of such examples to understand the underlying principles of regulation. Another emerging theme is how interactions between the nascent peptide and the exit tunnel of the ribosome can stall ribosomes by inactivating the PTC. Also here more examples of stalling structures would help to understand the prevalence of this phenomenon and its contribution to regulatory programs. Finally, the mechanism by which ribosome pausing affects nascent protein folding is poorly understood and would require development of new techniques to monitor protein folding in real time in atomic detail. The improvement of ribosome profiling techniques in conjunction with nascentome analysis and mass spectrometry, the increasing availability of high-resolution and high-throughput structural analysis of the stalling complexes, and development of biophysical methods that monitor the dynamics of ribosomes and nascent chains at single codon resolution will answer these questions and reveal yet another level of translation regulation acting as a “code-in-the-code” in cellular transcriptome.

## Author Contributions

All authors contributed to the writing of the manuscript. All authors have read and agreed to the published version of the manuscript.

### Conflict of Interest

The authors declare that the research was conducted in the absence of any commercial or financial relationships that could be construed as a potential conflict of interest.

## Abbreviations

TE: Translation efficiency – the number of protein molecules produced from one mRNA
ASL: tRNA anticodon stem-loop
PTC: Peptidyl transferase center
RNAP: RNA polymerase
SD: Shine-Dalgarno
